# A prospective observational study to assess the diagnostic accuracy of clinical decision rules for children presenting to emergency departments after head injuries (protocol): the Australasian Paediatric Head Injury Rules Study (APHIRST)

**DOI:** 10.1186/1471-2431-14-148

**Published:** 2014-06-13

**Authors:** Franz E Babl, Mark D Lyttle, Silvia Bressan, Meredith Borland, Natalie Phillips, Amit Kochar, Stuart R Dalziel, Sarah Dalton, John A Cheek, Jeremy Furyk, Yuri Gilhotra, Jocelyn Neutze, Brenton Ward, Susan Donath, Kim Jachno, Louise Crowe, Amanda Williams, Ed Oakley

**Affiliations:** 1 Department of Emergency Medicine, Royal Children’s Hospital, Flemington Rd, Parkville Vic 3052, Australia; 2 Murdoch Childrens Research Institute, Parkville, VIC, Australia; 3 Department of Paediatrics, Faculty of Medicine, Dentistry and Health Sciences, University of Melbourne, Melbourne VIC 3010, Australia; 4 National Trauma Research Institute, Prahan, VIC, Australia; 5 Bristol Royal Hospital for Children, Bristol, UK; 6 Academic Department of Emergency Care, University of the West of England, Bristol, UK; 7 University of Padova, Padova, Italy; 8 Princess Margaret Hospital for Children, Perth, Australia; 9 Royal Children's Hospital and Queensland Children's Medical Research Institute, Queensland University, Brisbane, Australia; 10 Women’s & Children’s Hospital, Adelaide, Australia; 11 Starship Hospital, Auckland, New Zealand; 12 Liggins Institute, University of Auckland, Auckland, New Zealand; 13 The Children’s Hospital at Westmead, Sydney, Australia; 14 Monash Medical Centre, Clayton, VIC, Australia; 15 Townsville Hospital, Townsville, Australia; 16 Mater Children’s Hospital, Brisbane, Australia; 17 Kidzfirst Middlemore Hospital, Auckland, New Zealand

**Keywords:** Head injury, Clinical decision rule, Computed tomography, Validation

## Abstract

**Background:**

Head injuries in children are responsible for a large number of emergency department visits. Failure to identify a clinically significant intracranial injury in a timely fashion may result in long term neurodisability and death. Whilst cranial computed tomography (CT) provides rapid and definitive identification of intracranial injuries, it is resource intensive and associated with radiation induced cancer. Evidence based head injury clinical decision rules have been derived to aid physicians in identifying patients at risk of having a clinically significant intracranial injury. Three rules have been identified as being of high quality and accuracy: the Canadian Assessment of Tomography for Childhood Head Injury (CATCH) from Canada, the Children’s Head Injury Algorithm for the Prediction of Important Clinical Events (CHALICE) from the UK, and the prediction rule for the identification of children at very low risk of clinically important traumatic brain injury developed by the Pediatric Emergency Care Applied Research Network (PECARN) from the USA. This study aims to prospectively validate and compare the performance accuracy of these three clinical decision rules when applied outside the derivation setting.

**Methods/design:**

This study is a prospective observational study of children aged 0 to less than 18 years presenting to 10 emergency departments within the Paediatric Research in Emergency Departments International Collaborative (PREDICT) research network in Australia and New Zealand after head injuries of any severity. Predictor variables identified in CATCH, CHALICE and PECARN clinical decision rules will be collected. Patients will be managed as per the treating clinicians at the participating hospitals. All patients not undergoing cranial CT will receive a follow up call 14 to 90 days after the injury. Outcome data collected will include results of cranial CTs (if performed) and details of admission, intubation, neurosurgery and death. The performance accuracy of each of the rules will be assessed using rule specific outcomes and inclusion and exclusion criteria.

**Discussion:**

This study will allow the simultaneous comparative application and validation of three major paediatric head injury clinical decision rules outside their derivation setting.

**Trial registration:**

The study is registered with the Australian New Zealand Clinical Trials Registry (ANZCTR)- ACTRN12614000463673 (registered 2 May 2014).

## Background

Children with clinically significant intracranial injuries require urgent identification to prevent further damage to the brain. Cranial computed tomography (CT) scans provide rapid and definitive identification of the presence or absence of intracranial injuries, and help guide subsequent management. Positive results allow early intervention and optimise outcomes whilst negative results are reassuring and may allow accelerated discharge and reduce unnecessary admissions.

However, cranial CT scans also have negative effects, particularly in children, who are more vulnerable to radiation-associated cell damage [1]. Radiation from cranial CT scans can cause lethal malignancies with higher risk in younger age groups [1-4]. Children may require sedation to allow imaging with consequent sedation-associated risks [5,6]. They also have resource implications for Emergency Departments (EDs) and the health system as a whole [7]. Despite this, the number of cranial CT scans performed for head injuries in children has increased in a number of countries [8-11]. This increase is likely due to a combination of easier access to CT scanners and more efficient technology and concern amongst physicians of being unable to reliably identify intracranial injury based solely on a child’s clinical condition. One way of increasing clinical sensitivity and specificity (i.e. minimising both missed clinically significant intracranial injuries and unnecessary investigations) is to develop and use clinical decision rules (CDRs).

CDRs help physicians with diagnostic and therapeutic decisions, and can be defined as decision making tools derived from original research (as opposed to a consensus-based clinical practice guideline) which incorporate three or more variables from the history, physical examination, or simple tests. These tools help clinicians cope with the uncertainty of medical decision making and improve their efficiency [12]. Several recent systematic reviews of existing paediatric head injury CDRs have been published [13-15]. The three CDRs of highest quality and accuracy [15] are the Canadian Assessment of Tomography for Childhood Head Injury (CATCH) from Canada [11], the Children’s Head Injury Algorithm for the Prediction of Important Clinical Events (CHALICE) from the UK [16] and the prediction rule for the identification of children at very low risk of clinically important traumatic brain injury developed by the Pediatric Emergency Care Applied Research Network (PECARN) from the USA [17]. All three CDRs were derived with high methodological standards using large multicentre data sets. However, they differ in key areas, including study population, predictor variables (based on mechanism of injury, clinical history, and clinical examination) (Table 1), inclusion and exclusion criteria (Table 2) and outcomes (including the terminology and definitions used) (Table 3). Most importantly the focus is different in each CDR. CATCH was derived to manage children with minor head injuries presenting within 24 hours, with specific inclusion criteria to be fulfilled before employing the CDR. CHALICE was derived for children with head injuries of all severities, presenting at any point after the injury. Both aim to identify children likely to have significant intracranial injury who warrant a cranial CT scan. PECARN’s CDR focuses on children with minor head injuries presenting within a 24 hour period and aims to identify patients unlikely to have a clinically important traumatic brain injury who can be safely discharged without a CT scan. In addition PECARN has derived different CDRs for children aged less than two years and children aged two years and older. The comparative performance accuracy (as assessed by sensitivity, specificity, negative predictive value and positive predictive value) for each CDR has been presented elsewhere [15]. CATCH and CHALICE CDRs suggest a dichotomous course of action (cranial CT scan/no cranial CT scan) although CATCH stratifies this risk into high and medium categories. The PECARN CDR defines a low risk population in whom cranial CT scans can routinely be obviated.

**Table 1:** **Comparison of predictor variables**[11,15-17]

**CATCH**	**CHALICE**	**PECARN <2 years**	**PECARN ≥2 years**
*Mechanism of injury*			
Dangerous mechanism of injury (eg MVC, fall from elevation ≥3 ft [≥0.91 m] or 5 stairs, fall from bicycle with no helmet).	High speed RTA as pedestrian, cyclist, occupant (>40 miles/h or >64 km/h).	Severe mechanism of injury (MVC with patient ejection, death of another passenger or rollover; pedestrian/bicyclist without helmet struck by motorized vehicle; falls >0.9 m; head struck by high impact object).	Severe mechanism of injury (MVC with patient ejection, death of another passenger or rollover; pedestrian/bicyclist without helmet struck by motorized vehicle; falls >1.5 m; head struck by high impact object).
	Fall of > 3 m in height.		
	High speed injury from projectile or object.		
*History*			
	Witnessed LOC > 5 min.	LOC ≥5 seconds.	Any/suspected LOC.
	Amnesia (antegrade or retrograde) >5 min.		
		Altered mental status.	Altered mental status.
		Not acting normally per parent.	
	≥3 vomits after head injury (discrete episodes).		History of vomiting.
	Suspicion of NAI.		
	Seizure in patient with no history of epilepsy.		
History of worsening headache.			Severe headache.
*Examination*			
GCS <15, 2 hr after injury.	GCS <14, or <15 if <1 yr.	GCS < 15	GCS < 15
Irritability on examination.	Abnormal drowsiness (in excess of that expected by examining doctor).	Other signs of altered mental status (agitation, somnolence, repetitive questioning, slow response to verbal communication)	Other signs of altered mental status (agitation, somnolence, repetitive questioning, slow response to verbal communication)
Suspected open or depressed skull fracture.	Suspicion of penetrating or depressed skull injury, or tense fontanelle.		
Any sign of basal skull fracture (eg haemotympanum, “raccoon” eyes, otorrhoea/rhinorrhoea of CSF, Battle’s sign).	Signs of basal skull fracture.	Palpable or unclear skull fracture.	Clinical signs of basilar skull fracture.
	Positive focal neurology.		
Large boggy haematoma of the scalp.	Presence of bruise, swelling or laceration > 5 cm if < 1 yr old.	Occipital, parietal or temporal scalp haematoma.	

Reproduced from Lyttle M, *et al.*[15] Copyright 2012, with permission from BMJ Publishing Group Ltd.

In each of the three clinical decision rules (CDRs) the absence of all of the above predictor variables indicates that cranial computed tomography is unnecessary.

Note: while the predictor variables are reproduced verbatim, the order in which the variables from each CDR are presented has been altered to facilitate comparison.

*CATCH* Canadian Assessment of Tomography for Childhood Head Injury.

*CHALICE* Children’s Head Injury Algorithm for the Prediction of Important Clinical Events.

*PECARN* Pediatric Emergency Care Applied Research Network.

*MVC* Motor vehicle crash.

*RTA* Road traffic accident.

*LOC* Loss of consciousness.

*NAI* Non-accidental injury.

*GCS* Glasgow Coma Score.

*CSF* Cerebrospinal fluid.

**Table 2:** **Comparison of inclusion and exclusion criteria**[11,15-17]

	**Inclusion criteria**	**Exclusion criteria**
**CATCH**	*All of the following:*	*Any of:*
	• Blunt trauma to head resulting in witnessed LOC/disorientation, definite amnesia, persistent vomiting (>1 episode), persistent irritability (in children <2 yrs)	• Obvious penetrating skull injury
		• Obvious depressed fracture
		• Acute focal neurologic deficit
		• Chronic generalized developmental delay
		• Head injury secondary to suspected child abuse
	• Initial GCS in ED ≥13 as determined by treating physician	• Returning for reassessment of previously treated head injury
	• Injury within the past 24 hours.	• Patients who were pregnant
**CHALICE**	Any history or signs of injury to the head.	Refusal to consent
**PECARN**	Present within 24 hours of head injury.	*Any of:*
		• Trivial head injury (defined by ground level fall, walking/running into stationary object, no signs or symptoms of head trauma except scalp abrasions and lacerations).
		• Penetrating trauma
		• Known brain tumour
		• Pre-existing neurological disorder complicating assessment
		• Neuro-imaging at another hospital before transfer
		• Patient with ventricular shunt*
		• Patient with bleeding disorder*
		• GCS < 14*

Reproduced from Lyttle M, *et al.*[15] Copyright 2012, with permission from BMJ Publishing Group Ltd.

*CATCH* Canadian Assessment of Tomography for Childhood Head Injury.

*CHALICE* Children’s Head Injury Algorithm for the Prediction of Important Clinical Events

*PECARN* Pediatric Emergency Care Applied Research Network.

*GCS* Glasgow Coma Score.

*LOC* Loss of consciousness.

*ED* emergency department.

*enrolled but being analysed separately, not used in clinical decision rule derivation.

**Table 3:** **Comparison of outcomes**[11,15-17]

	***Primary outcome***	***Secondary outcomes***
CATCH	Need for neurological intervention, defined as death within 7 days secondary to the head injury or need for any of the following within 7 days: craniotomy, elevation of skull fracture, monitoring of intracranial pressure, insertion of endotracheal tube for the management of head injury	Brain injury on CT, defined as any acute intracranial finding revealed on CT attributable to acute injury, including closed depressed skull fracture (depressed past the inner table) and pneumocephalus but excluding non-depressed skull fractures and basilar skull fractures
CHALICE	Clinically significant intracranial injury (CSII), defined as death as a result of head injury, requirement for neurosurgical intervention, marked abnormality on CT (any new, acute, traumatic intracranial pathology as reported by consultant radiologist, including intracranial haematomas of any size, cerebral contusion, diffuse cerebral oedema and depressed skull fractures)	Presence of skull fracture Admission to hospital
PECARN	Clinically important traumatic brain injury (ciTBI), defined as death from TBI, neurosurgical intervention for TBI (intracranial pressure monitoring, elevation of depressed skull fracture, ventriculostomy, haematoma evacuation, lobectomy, tissue debridement, dura repair, other), intubation of more than 24 h for TBI or hospital admission of 2 nights or more for TBI* in association with TBI on CT**	None

Reproduced from Lyttle M, *et al.*[15] Copyright 2012, with permission from BMJ Publishing Group Ltd.

*Admission for persistent neurological symptoms or signs such as persistent alteration in mental status, recurrent emesis due to head injury, persistent severe headache or ongoing seizure management.

**Intracranial haemorrhage or contusion, cerebral oedema, traumatic infarction, diffuse axonal injury, shearing injury, sigmoid sinus thrombosis, midline shift of intracranial contents or signs of brain herniation, diastasis of the skull, pneumocephalus, skull fracture depressed by at least the width of the table of the skull.

*CATCH* Canadian Assessment of Tomography for Childhood Head Injury.

*CHALICE* Children’s Head Injury Algorithm for the Prediction of Important Clinical Events.

*PECARN* Pediatric Emergency Care Applied Research Network.

*CT* computed tomography.

PECARN’s is the only CDR which has been internally [17] and externally [18] validated. A CATCH validation study has been performed in the derivation setting though results are only available in abstract form at present [19]. Recently the three CDRs have been prospectively validated in the same cohort of 1,009 children presenting to an urban medical center with a designated paediatric ED in the United States. This study showed that baseline physician ordering practice and PECARN outperformed the other CDRs. However, the study population did not reflect the exact population for which each rule was originally developed and the study was underpowered to determine narrow confidence intervals for rare but critically important events [20].

We propose to validate and compare the accuracy of the CATCH, CHALICE and PECARN CDRs using prospectively collected data from 20,000 patients in a multicentre setting in Australia and New Zealand, i.e. outside the countries where these CDRs were derived, and compare their performance against that of our current practice. Triggers for cranial CT use by clinicians in paediatric EDs in Australia and New Zealand are different from the triggers developed in CATCH, CHALICE and PECARN [21]. This study will also help determine which CDR is best suited for use in the Australian and New Zealand setting before incorporating them into local practice.

## Methods/design

### Aims

**Figure 1:**
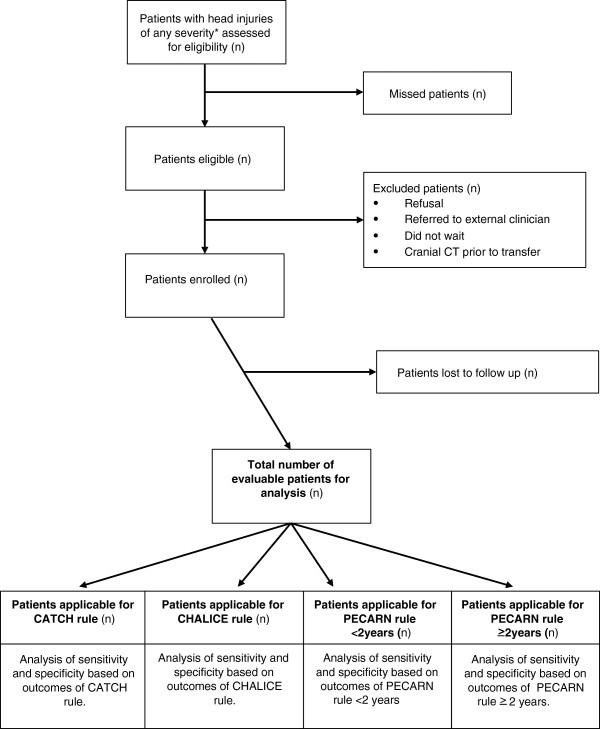
**Algorithm for patient eligibility and analysis.** *Head injuries not including trivial facial injuries defined as a ground level fall or walking or running into an object with no signs or symptoms of injury other than facial abrasions or lacerations below the eyebrows. CT computed tomography. LTFU lost to follow up. CHALICE Children’s Head Injury Algorithm for the Prediction of Important Clinical Events. CATCH Canadian Assessment of Tomography for Childhood Head Injury. PECARN Pediatric Emergency Care Applied Research Network.

The primary aim of this study is to determine the performance accuracy of the three major international paediatric head injury CDRs (CATCH, CHALICE and PECARN) when applied to a prospective multicentre population of consecutive children presenting with head injury to 10 EDs in Australia and New Zealand. This will allow the comparative external validation of the CDRs outside their derivation settings (Figure 1).

### Design

This is a multi-centre prospective observational study of consecutive children presenting with head injuries to paediatric EDs. All data points necessary for analysis including predictor variables and outcome data for the three clinical rules under investigation (Tables 1, 2 and 3) will be collected for all patients but treating clinicians will manage patients as per their usual practice. The study has been registered with the Australian New Zealand Clinical Trials Registry (ACTRN12614000463673). The study follows the STAndards for the Reporting of Diagnostic accuracy studies (STARD) guidelines [22].

### Setting

The study is taking place at 9 tertiary paediatric EDs, and 1 large combined adult and paediatric ED in Australia and New Zealand. These centres are members of the Paediatric Research in Emergency Departments International Collaborative (PREDICT) [23]: in New Zealand Kidz First Children’s Hospital, Auckland, and Starship Children’s Hospital, Auckland; in Australia Monash Medical Centre, Clayton, VIC, Children’s Hospital at Westmead, Sydney, NSW, Royal Children’s Hospital, Melbourne, VIC, Royal Children’s Hospital, Brisbane, QLD, Mater Children’s Hospital, Brisbane, QLD, Princess Margaret Hospital for Children, Perth, WA, Women’s & Children’s Hospital, Adelaide, SA, and Townsville Hospital, Townsville, QLD. The annual paediatric census of the 10 participating EDs is >400,000. The central site for the study is the Murdoch Children’s Research Institute, which is affiliated with the Royal Children’s Hospital Melbourne.

### Inclusion criteria

Patients less than 18 years of age with head injuries of all severities irrespective of length of time from injury to presentation will be included. The definition of head injury does not include patients who have sustained a trivial facial injury (ground level fall or walking or running into an object with no signs or symptoms of injury other than facial abrasions or lacerations below the eyebrows).

### Exclusion criteria

We will exclude patients and families who refuse to participate, are being referred directly from ED triage to a general practitioner or other external provider (i.e. not seen in the ED), or who do not wait to be seen. We will exclude from analysis patients with neuroimaging prior to transfer (Figure 1). Individual exclusion criteria (relevant to each CDR (Table 2)) will be applied during analysis.

### Primary outcome measure

Primary outcome will be the performance accuracy (sensitivity, specificity, negative predictive value (NPV), and positive predictive value (PPV)) of each CDR in identifying rule specific outcomes (Table 3) when applied to those patients who meet the individual inclusion and exclusion criteria (Table 2).

### Secondary outcome measures

1. Rate of clinically important traumatic brain injury (ciTBI) [17] and clinically significant intracranial injury (CSII) [16] in the study population.

2. Rate of neurosurgical intervention in the study population.

3. Rate of cranial CT use in the study population.

4. Number of missed ciTBI and CSII in the study population.

5. Characteristics of missed significant intracranial injuries that would have been identified by the application of each CDR to the study population.

6. Number of extra cranial CT scans that would be performed by applying each CDR.

7. Sensitivity, specificity, NPV and PPV of PECARN in identifying traumatic brain injury on cranial CT.

8. Diagnostic accuracy of each of the CDRs when applied to those patients attending with head injury who do not meet the specific individual inclusion and exclusion criteria.

9. Rule performance in patients with bleeding diathesis, ventriculoperitoneal shunt, non-accidental injuries and pre-existing neurological conditions.

10. Economic evaluation of financial savings or burden of implementing each CDR.

11. Rate of prolonged symptoms following a non-severe head injury.

### Patient recruitment, study procedure and data collection

Patients with head injuries will be identified at the ED triage desk using electronic alerts and visual reminders for patients who receive a head injury type injury code. Triage nurses will attach a clinician study clinical report form (CRF) to the patient record. Patients will be enrolled in the study by the treating clinician. Verbal consent for participation will be sought and documented by the treating clinician; consent for participation will include permission to telephone families 14–90 days after the ED visit for follow-up. Consent will be sought at the time of the initial ED visit. Should the parent or guardian of the child not be available at that time, we will seek consent for involvement in the study either during the in-patient stay (where admitted) or at the time of telephone follow up (where discharged from ED). Identification of missed eligible patients will be undertaken by the research assistant in each participating centre through a review of the daily ED attendance record.

Data collected by the ED treating clinicians will include the predictor variables from the three CDRs (CATCH [11], PECARN [17], CHALICE [16]). The initial ED assessment data will be documented prior to management decisions.

A separate CRF will be completed by the site research assistant during the hospital stay (in admitted patients) or after ED discharge (in those patients discharged direct from ED) once outcome data are available. It will collect the following parameters: detailed demographics, time lines (times of triage, clinician evaluation, ED and hospital discharge), ED observation and duration of observation, admission status and duration of admission, intensive care admission, intubation and ventilation and duration of ventilation, imaging and results, neurosurgical interventions and mortality.

The telephone follow-up to screen for possible initially missed intracranial injuries will be completed by the research assistant or the site physician investigators 14–90 days after the injury if no cranial CT is performed. Data on ongoing signs and symptoms, neuroimaging, admission and neurosurgery will be elicited. Six contact attempts will be made. If more than 90 days have elapsed from the time of injury, or if there have been six failed contact attempts, the patient follow up will be regarded as unsuccessful and the patient deemed lost to follow up.

All study materials have been piloted at a single site (Royal Children’s Hospital Melbourne) [23]; modification of the materials to comply with local patient flow and administrative requirements have been assessed and approved by the study steering committee.

CRFs will be de-identified after all data points have been completed and any data queries have been addressed. Data collation and analysis will take place at the central study site (Murdoch Childrens Research Institute, Melbourne).

All participating clinicians (physicians and nurse practitioners) at all sites receive formal training in the completion of the clinician CRF prior to the commencement of the study. Research assistants collecting data on the accompanying CRFs undergo formal training at the central site prior to the commencement of the study. Standardised teaching materials have been created and provided to participating sites. The study coordinator will ensure that all staff have received appropriate orientation and training and will ensure compliance with study protocol through site visits. Investigators and research assistants are not blinded to the results of the collected outcome data.

### Determination of outcome

Patient outcome will be determined by:

1. Consultant radiologist reports of CTs.

2. Operative reports for those who required neurosurgical intervention.

3. Review of medical record for the duration of admission and secondary outcomes.

4. Structured telephone follow up at 14–90 days post injury for patients discharged without neuroimaging.

5. Patients for whom final outcome data are not available will be excluded from data analysis.

This process will permit the identification of the presence and extent of injury allowing classification as per the definitions of each head injury CDR.

### Definitions

CDR specific definitions of inclusion and exclusion criteria, predictor variables and outcomes are set out in Tables 1, 2 and 3.

Further definitions used:

**ED observation:** Ongoing clinical assessment and observation of the patient in ED for less than 6 hours post initial clinical assessment.

**Admission:** Transfer from ED to a hospital inpatient unit (including short stay, observation, or intensive care unit) for longer than 6 hours.

**Neurosurgical interventions will be categorised based on operative reports into the following categories:** Dura repair of cerebrospinal fluid leaks, skull fracture elevation, haematoma drainage, intracranial pressure (ICP) monitoring, lobectomy, tissue debridement, ventriculostomy, other.

**Head imaging (CT and magnetic resonance imaging) will be categorised as follows based on reports by consultant radiologists:** Cerebellar haemorrhage, cerebral contusion, cerebral oedema, cerebral haemorrhage, intracerebral haematoma, diastasis of the skull, extradural/epidural haematoma, extra-axial haematoma, intraventricular haemorrhage, midline shift/shift of brain structures, pneumocephalus, skull fracture (and depth of depression), subarachnoid haemorrhage, subdural haematoma, traumatic infarction.

## Statistical methods

When applying each CDR, items will be scored as present, absent or unknown. Sensitivity, specificity, negative predictive value (NPV), and positive predictive value (PPV) of each of the CDRs will be calculated using the definitions and parameters set out in the derivation studies as published [11,16,17]. In addition, the two CDRs limited to minor head injuries (CATCH and PECARN) will also be applied to patients of all head injury severities to assess their performance in this extended patient group. Likewise, the CHALICE CDR, though derived for all severities of head injury, will undergo separate analysis in minor head injury to allow comparison of performance accuracy of the three CDRs in that population. Performance accuracy will also be calculated in patient subgroups including but not restricted to patients with bleeding diathesis and ventriculoperitoneal shunts. Rates of secondary outcomes such as cranial CT, neurosurgical intervention, ciTBI and CSII and missed ciTBI and CSII will be calculated. Key percentages will be presented with 95% confidence intervals. Data will be entered using Epidata (The Epidata Association, Odense, Denmark) and analysed using Stata 12 (Statacorp, College Station, Texas, USA).

### Sample size and power calculation

In deriving a sample size for patient subgroups we extrapolated from the PECARN data as it is the only CDR which differentiates between children aged less than two years and children aged two years and older [17].

Based on PECARN’s ciTBI rate of 1%, and the ability to determine the sensitivity and specificity of the CDRs to a precision level of between 94% and 100%, we determined that we would require 10,000 patients to be enrolled in our study in order to maintain the precision for the two subgroups in the PECARN CDR, children aged less than two years and children aged two years and older (i.e. 5,000 children in each age sub-group). Previous retrospective research of children diagnosed with a head injury, conducted at Royal Children’s Hospital Melbourne, had identified a 1:1 ratio between children aged less than two years and those aged two years or greater [24]. After an analysis of the first 1,000 patients enrolled in the APHIRST study [23] this premise was found to be incorrect and in the prospectively enrolled patients the true ratio of children less than 2 years presenting with a head injury to children 2 years of age or older presenting with a head injury was 1:4. Therefore, to preserve the precision of the study in the younger age group of children for the PECARN CDR the sample size was recalculated to 20,000 children. Table 4 illustrates the precision that would be achieved (using 95% confidence intervals) based on these assumptions for several different plausible values for sensitivity for the outcomes (i) ciTBI (ii) need for neurosurgery and (iii) brain injury on CT (as based on PECARN data [17]).

**Table 4:** **Projected sensitivity for outcomes of clinically important traumatic brain injury (ciTBI), need for neurosurgery and brain injury on computed tomography (CT) based on PECARN data**[17]

**Outcome**	**Number of patients predicted**	**Projected performance of CDR in predicting outcome (sensitivity)**	**Sensitivity%**	**95% confidence interval**
ciTBI	50	50/50	100	93-100
	50	49/50	98	89-100
	50	48/50	96	86-99.5
	50	47/50	94	83-99
Need for neurosurgery	30	30/30	100	88-100
	30	29/30	96.5	83-100
Brain injury on CT	300	300/300	100	98.8-100
	300	290/300	97	94-98

*CDR* clinical decision rule.

### Ethical issues and consent

In this observational non-interventional study parental verbal consent and participant verbal assent (for patients deemed capable to understand and appropriately answer questions) will be obtained for all patients; it will include permission to conduct a follow-up telephone call to determine outcome. Delayed consent at the time of the phone call if necessary has been approved for patients not enrolled during the initial ED visit. Ethics approval has been granted at all 10 study sites.

Patients who refuse consent or withdraw will continue to be managed as per the treating clinician.

As this is an observational study we are not anticipating adverse events.

## Limitations

Ideally, all patients with head injuries would receive a cranial CT to determine the presence or absence of significant intracranial injuries. However, this would expose a large number of patients to unnecessary CTs and the associated cancer related risks; therefore, similar to the methodology used in the derivation and validation studies for CATCH [11] and PECARN [17] this study relies on patient follow up by telephone. In doing so we will establish whether a relevant outcome has occurred or not.

CT rates in Australia and New Zealand may be lower [23,25] than in North America and as reported for the CATCH and PECARN studies [8,10,11,17] and higher than the baseline rates reported from the United Kingdom in the CHALICE study [16]. This highlights one of the potential key strengths of this study as it tests the CDRs in a setting different to that in which each one was derived. Finally, while we were provided with copies of the telephone follow up questionnaires used by the CATCH and PECARN investigators (personal communication, Dr Martin Osmond and Dr Nathan Kuppermann) we reconstructed the predictor variables for the three CDRs solely from the published papers [11,16,17]. This may have introduced an element of interpretation in terms of the most precise wording to be used in a clinical emergency setting.

## Discussion

This study will allow the simultaneous comparative application and validation of three major paediatric head injury clinical decision rules outside their derivation setting. In addition to a high recruitment rate, the study will depend on high follow up rates to ensure that our results accurately represent the whole population of children presenting with head injuries.

## Time plan

We have so far recruited more than 10,000 of the planned 20,000 patients. We will complete recruitment by the end of 2014.

## Abbreviations

CHALICE: Children’s head injury algorithm for the prediction of important clinical events; CATCH: Canadian assessment of tomography for childhood head injury; PECARN: Pediatric emergency care applied research network; CT: Computed tomography; CSII: Clinically significant intracranial injury; ciTBI: Clinically important traumatic brain injury; CDR: Clinical decision rule; ED: Emergency Department; NPV: Negative predictive value; PPV: Positive predictive value.

## Competing interests

None of the authors have any competing interests arising from this research.

## Authors’ contributions

FEB was responsible for identifying the research question and the design of the study. FEB, MDL and EO were responsible for refining the design and developing the research protocol. All authors have contributed to the development of the protocol, the implementation of the study at participating sites and the enrolment of patients. FEB was responsible for the drafting of this paper. All authors provided comments on the drafts and have read and approved the final version. FEB takes responsibility for the manuscript as a whole.

## Pre-publication history

The pre-publication history for this paper can be accessed here:

http://www.biomedcentral.com/1471-2431/14/148/prepub
